# ﻿*Lysimachiafenghwaiana* (Primulaceae), a new species from Hunan Province, China

**DOI:** 10.3897/phytokeys.220.99556

**Published:** 2023-02-24

**Authors:** Hai-Fei Yan, Jia-Xiang Li, Tong-Jian Liu, Gang Hao

**Affiliations:** 1 Key Laboratory of Plant Resources Conservation and Sustainable Utilization, South China Botanical Garden, Chinese Academy of Sciences, Guangzhou 510650, China South China Botanical Garden, Chinese Academy of Sciences Guangzhou China; 2 South China National Botanical Garden, Guangzhou 510650, China South China National Botanical Garden Guangzhou China; 3 College of Forestry, Central South University of Forestry and Technology, Changsha 410004, China Central South University of Forestry and Technology Changsha China; 4 College of Life Sciences, South China Agricultural University, Guangzhou 510642, Guangdong, China South China Agricultural University Guangzhou China

**Keywords:** central China, Ericales, flora, morphological features, taxonomy

## Abstract

A new species, *Lysimachiafenghwaiana* G.Hao & H.F.Yan (Primulaceae), from Hunan Province, China, is described and illustrated. This new species belongs to Lysimachiasubgen.Lysimachiasect.Nummularia and is morphologically similar to *L.crista-galli* and *L.carinata*, but is distinctive in its leaf shape and arrangement of flowers. It can be further distinguished from *L.crista-galli* by the absence of calyx lobule spur, and from *L.carinata* by the black glandular striates in the corolla lobes, rather than punctate.

## ﻿Introduction

*Lysimachia* L. is one of the largest genera of Primulaceae, and it had been known to comprise about 180 species worldwide (Chen et al. 1989; Hu and Kelso 1996). As a whole, it is almost cosmopolitan, with the greatest diversity of species occurring in southwest China, especially in Sichuan, Guizhou and Yunnan Provinces. As a result of various molecular phylogenetic analyses over the past two decades, the alignment of the genus has been largely modified, with expansion to include some monotypic or small genera, for example, *Anagallis* L., *Glaux* L., *Pelletiera* A. St.-Hil. and *Trientalis* L. (Hao et al. 2004; Banfi et al. 2005; Anderberg et al. 2007; Manns and Anderberg 2009; Yan et al. 2018). The total number of species of *Lysimachia* has accordingly increased to approximately 250 (Yan et al. 2018).

Some new *Lysimachia* species have been continually described in recent years, mainly from the areas of central and south-western China (e.g. Zhou et al. 2015; Yan et al. 2017; Huang et al. 2019; Huang et al. 2020; Mou et al. 2020; Ju et al. 2021; Ke et al. 2021). During a field expedition conducted in Pingjiang County, Yueyang City, Hunan Province, in July 2021, a new taxon of *Lysimachia* was found, which is described here as a species new to science named *L.fenghwaiana* G.Hao & H.F.Yan, affiliated to Lysimachiasubgen.Lysimachia sect. Nummularia (Gilib.) Klatt.

## ﻿Materials and methods

Historical taxonomic literature has been consulted (e.g. Handel-Mazzetti 1928; Chen and Hu 1979; Chen et al. 1989; Hu and Kelso 1996) to infer similar species and relatedness. The new species was examined in the field and at the herbarium, and measurements of morphological features were conducted with fresh specimens. Particularly, flowers were dissected and photographed. Morphological comparison with related species was performed based on living plants and specimens from IBSC, PE, IBK and CSFI and from the images of specimens from the JSTOR Global Plants (http://plants.jstor.org/). The conservation status of the new species was assessed following the guidelines for using the IUCN Red List Categories and Criteria (IUCN Standards and Petitions Committee 2022).

## ﻿Taxonomic treatment

### ﻿ Lysimachia fenghwaiana

Taxon classificationPlantaeEricalesPrimulaceae

G.Hao & H.F.Yan sp. nov.

97C37148-213C-5359-A0FC-80BD4F3E4081

urn:lsid:ipni.org:names:77314718-1

Figs 1 , 2 , 3

#### Type.

China. Hunan Province, Yueyang City, Pingjiang County, Lutou Forest Farm, 28°32'N, 113°55'E, alt. 421 m, 22 May 2022, *Hai-Fei Yan and Chun-Lai Zhang Yan2022050* (holotype: IBSC! barcode IBSC0895001).

#### Diagnosis.

*Lysimachiafenghwaiana* is most similar to *L.crista-galli* Pamp. & Hand. -Mazz. and *L.carinata* Y.I.Fang & C.Z.Cheng, but is different in its leaf shape and arrangement of flowers. It further differs from *L.crista-galli* in the absence of calyx lobule spur, and differs from *L.carinata* in the black glandular striates in the corolla lobes (vs. punctate).

#### Description.

Herbs perennial, 20 to 70 cm tall. Stems erect, later arched to reclined, simple or shortly branched, initially covered with rust-coloured multicellular hairs, glabrescent. Leaves opposite; petioles 0.6–1.1 cm long, sparsely strigillose; blades broadly ovate, 1.2–2.8 × 0.8–1.8 cm, sparsely strigillose abaxially, densely short black glandular striate, base broadly cuneate, margin subentire, apex subacute to obtuse; midrib sunken abaxially, prominent abaxially when dry, secondary veins 3 or 4 pairs, veinlets inconspicuous. Flowers solitary or paired, in axis of apical leaves; pedicel 1–1.8 cm, glandular pubescent. Calyx 5-parted, green, lobes lanceolate, 5–6 mm long, abaxially cristate; crest widest at base, ca. 2 mm, black glandular striate, apex acute. Corolla yellow, tube ca. 2 mm long, lobes elliptic-lanceolate, ca. 11 × 4 mm, densely black glandular striate, apex obtuse. Stamens 5, filaments 3.2–3.5 mm long, connate basally into a tube, tube part 3.8–4.0 mm long, adnate to corolla tube, anthers oblong, ca. 1.8 mm long, dorsifixed, opening by lateral slits. Ovary ovoid, 1 mm long, glabrous, style ca. 7 mm long, stigma capitate. Capsules subspherical, ca. 5 mm in diameter, glabrous.

#### Distribution and habitat.

The new species is currently known only from the type locality in Hunan Province, i.e. Lutou Forest Farm in Pingjiang County, Yueyang City. It grows at the edge of secondary mixed-evergreen forests, or under open forest on the hillside, at an altitude of ca. 400–450 m a.s.l.

#### Phenology.

Flowering from May to June, fruiting from July to August.

#### Etymology.

The new species is named in honour of Prof. Feng-Hwai Chen, a Chinese plant taxonomist and horticulturist, who devoted all his life to the development of botanical gardens in China and made considerable contributions to the study of Primulaceae and Asteraceae.

#### Local name.

Simplified Chinese: 芦头过路黄; Chinese Pinyin: Lútou Guò Lù Huáng. “Lútou” means the flowers of *Phragmitescommunis* Trin. (Poaceae), which abundantly occurs locally. “Guò Lù Huáng” means plants of *Lysimachia*.

#### Conservation status.

Based on our field investigations in Yueyang City and adjacent areas (e.g. Hubei and Guangxi Provinces) in the past ten years, only one population with ca. 1000 individuals of the new species has been found in an area of 10 km^2^ in Lutou Forest Farm, Pingjiang County, Yueyang City. Moreover, the habitats are under threat from road construction and timber harvesting. Therefore, the conservation status of the new species is assessed as Critically Endangered (CR) (B2a & bi, iii), according to the guidelines for using the IUCN Red List Categories and Criteria (IUCN Standards and Petitions Committee 2022).

#### Additional specimens examined

**(paratypes).** China. The same locality as holotype, 25 July 2021, *Hai-Fei Yan et al. Yan2021069* (IBSC!); The same locality as holotype, 4 June 2012, under forest, alt. ca. 500 m, *Jiaxiang Li et al. 1855* (CSFI! barcode CSFI069374).

#### Relationship with related species.

**Figure 1.**
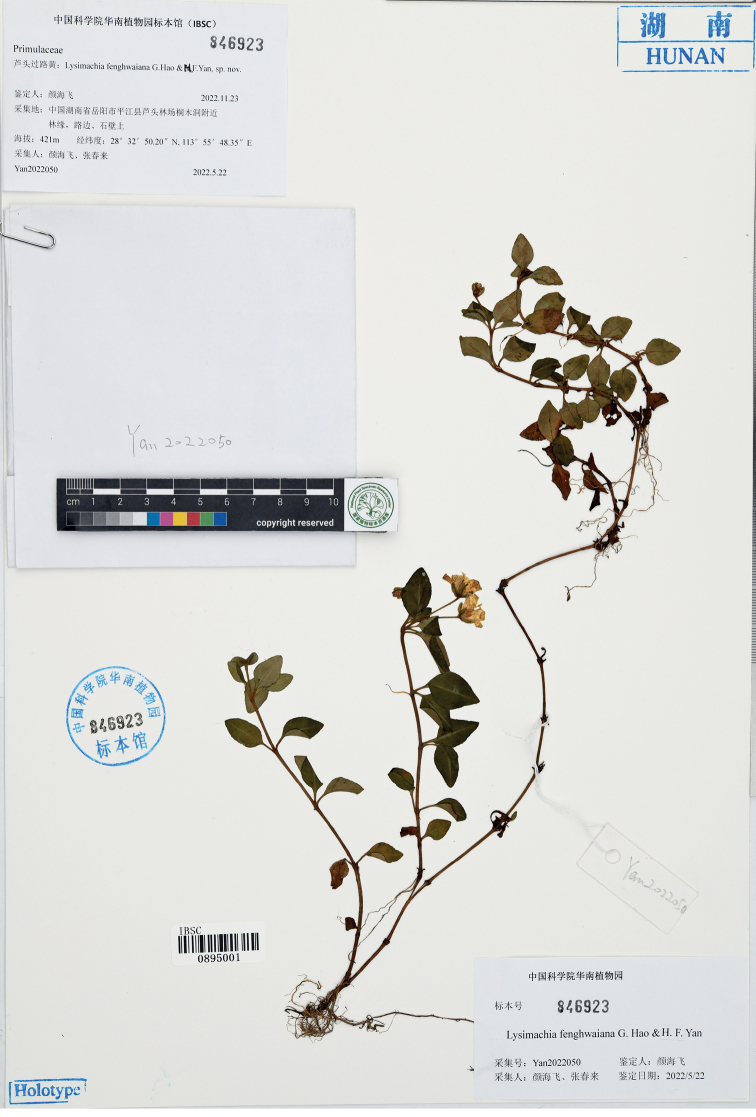
Holotype of *Lysimachiafenghwaiana* G.Hao & H.F.Yan, sp. nov. (*Hai-Fei Yan and Chun-Lai Zhang Yan2022050*, IBSC barcode IBSC0895001).

**Figure 2.**
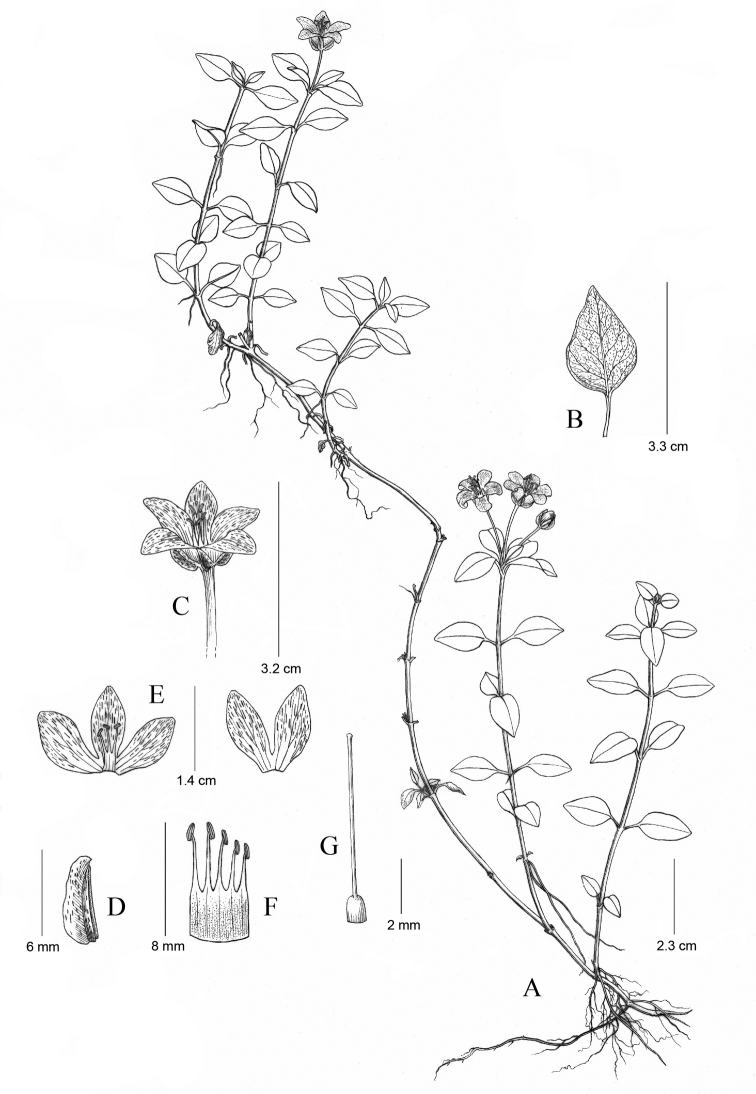
*Lysimachiafenghwaiana* G.Hao & H.F.Yan, sp. nov. **A** habit **B** abaxial surface of leaf **C** flower **D** calyx-lobe showing crest ridge **E** dissected corolla **F** stamens **G** pistil. Drawn by Yun-Xiao Liu from the holotype.

Based on the classification of *Lysimachia* by Handel-Mazzetti (1928) and Chen and Hu (1979), the new species clearly belongs to LysimachiasubgenusLysimachia sect. Nummulariaser.Drymarifoliae Hand.-Mazz., which is characterised by filaments connate into a tube, adnate to the base of corolla tube; anthers shorter than filaments, opening by lateral slits; and plants producing coloured punctate or striate glands. Amongst this series, approximately six species constitute a group, highlighted by the calyx with crested ridges (Handel-Mazzetti 1928; Chen et al. 1989; Zhou et al. 2015). The new species belongs to the group by having a crested calyx (Figs 1D, 2G) and is morphologically similar to *L.crista-galli* and *L.carinata*, but is distinctive in its flowers occurring in the axis of the apical leaves, rather than in the axis of the middle and upper leaves in the latter two species. Further, from *L.crista-galli*, it differs in its cuneate leaf base and absence of corolla lobule spur (vs. leaf base cordate and calyx lobule spur present in *L.crista-galli*); and from *L.carinata*, it differs by the shape of leaf lamina and corolla, i.e striate in *L.crista* (vs. punctate in *L.carinata*) (see Table 1).

**Figure 3.**
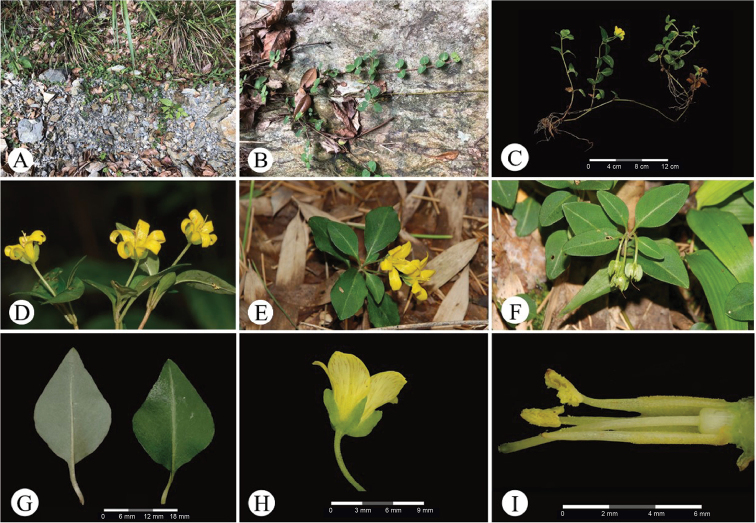
Living plant of *Lysimachiafenghwaiana* G.Hao & H.F.Yan, sp. nov. **A** habitat **B, C** habit **D, E** flowering plant **F** fruiting plant **G** leaves on abaxial (left) and adaxial (right) surfaces **H** flower (lateral view) **I** stamens and pistil (partial). Photographed by Jia-Xiang Li and Hai-Fei Yan.

**Table 1.** Main morphological differences between *Lysimachiafenghwaiana* and two similar species.

Features	*L.fenghwaiana*	*L.crista-galli*	*L.carinata*
Lamina shape	broadly ovate, base rounded to truncate, apex subacute to obtuse	broadly ovate to suborbicular, base cordate, apex subacute to obtuse	broadly ovate to ovate, base rounded to truncate, apex acute to acuminate
Lamina glands’ type	short striate	mix of both striate and punctate	punctate
Arrangement of flowers	solitary or paired in axis of apical leaves	solitary, in axis of middle and upper leaves	solitary or paired, in axis of middle and upper leaves
Calyx lobule spur	Absent	Present	absent
Corolla lobule glands	densely striate	densely striate	punctate

Whether the development of the crest to the calyx lobes, i.e. the winged keel of the calyx lobes, is a synapomorphy and those species constitute a monophyletic group in *Lysimachia* is uncertain, and further phylogenetic analysis should be undertaken to resolve this issue.

## Supplementary Material

XML Treatment for Lysimachia fenghwaiana

## References

[B1] Anderberg AA Manns U Källersjö M (2007) Phylogeny and floral evolution of the Lysimachieae (Ericales, Myrsinaceae). Evidence from *ndh*F sequence data. Willdenowia 37(2): 407–421. 10.3372/wi.37.37202

[B2] Banfi E Galasso G Soldano A (2005) Notes on systematics and taxonomy for the Italian vascular flora 1. Atti della Societa Italiana di Scienze Natturali e del Museo Civico di Storia Naturale di Milano 146: 219–244.

[B3] Chen FH Hu CM (1979) Taxomomic and phytogeographic studies on Chinese species of *Lysimachia*. Zhiwu Fenlei Xuebao 17: 21–53.

[B4] Chen FH Hu CM Fang YY Zheng CZ (1989) *Lysimachia*. In: Chen FH, Hu CM (Eds) Flora Reipublicae Popularis Sinicae (Vol. 59 (1)). Science Press, Beijing, 3−133.

[B5] Handel-Mazzetti H (1928) A revision of the Chinese species of *Lysimachia*. Notes from the Royal Botanic Garden Edinburgh 77: 51–122.

[B6] Hao G Yuan YM Hu CM Ge XJ Zhao NX (2004) Molecular phylogeny of *Lysimachia* (Myrsinaceae) based on chloroplast *trn*L-F and nuclear ribosomal ITS sequences. Molecular Phylogenetics and Evolution 31(1): 323–339. 10.1016/S1055-7903(03)00286-0 15019628

[B7] Hu CM Kelso S (1996) Primulaceae. In: Wu ZY Raven PH (Eds) Flora of China (Vol.15). Science Press, Beijing, and Missouri Botanical Garden Press, St Louis, 99–185.

[B8] Huang YF Dong LN Xu WB (2019) *Lysimachiafanii*, a new species of Primulaceae from limestone area of Guangxi, China. PhytoKeys 130: 75–84. 10.3897/phytokeys.130.34655 31534396 PMC6728382

[B9] Huang RZ Liao M Han W Yang YZ Zhou MY Feng HH Tang GD (2020) *Lysimachiadaqiaoensis* (Primulaceae), a new cave species from Guangdong, China. Phytotaxa 430: 41–45. 10.11646/phytotaxa.430.1.6

[B10] IUCN Standards and Petitions Committee (2022) Guidelines for using the IUCN Red List categories and criteria. Version 15.1: Prepared by the Standards and Petitions Committee in July 2022.

[B11] Ju WB Deng HN Xu B He XJ Gao XF (2021) *Lysimachiaxuyongensis* (Primulaceae), a new species from Sichuan, China. Phytotaxa 525(1): 59–64. 10.11646/phytotaxa.525.1.7

[B12] Ke ZW Gan QL Li XW (2021) *Lysimachiabrevianthera* (Primulaceae), a new species from the Daba Mountains in Hubei and Shaanxi, China. Annales Botanici Fennici 58(4–6): 253–258. 10.5735/085.058.0410

[B13] Manns U Anderberg AA (2009) New combinations and names in *Lysimachia* (Myrsinaceae) for species of *Anagallis*, *Pelletiera* and *Trientalis*. Willdenowia 39(1): 49–54. 10.3372/wi.39.39103

[B14] Mou C Wu Y Xiang L Xiang XM Zhang DG (2020) *Lysimachiaxiangxiensis* (Primulaceae), a new species from limestone area in Hunan Province, central China. PhytoKeys 140: 23–32. 10.3897/phytokeys.140.47995 32148429 PMC7052036

[B15] Yan HF Xu Y Zhu ZM Hu CM Hao G (2017) *Lysimachiasinopilosa* (Primulaceae), a New Species from Yunnan, China. Annales Botanici Fennici 54(1–3): 45–48. 10.5735/085.054.0308

[B16] Yan HF Zhang CY Anderberg AA Hao G Ge XJ Wiens JJ (2018) What explains high plant richness in East Asia? Time and diversification in the tribe Lysimachieae (Primulaceae). The New Phytologist 219(1): 436–448. 10.1111/nph.15144 29663397

[B17] Zhou JJ Yu XL Deng YF Yan HF Lin ZL (2015) *Lysimachiahuangsangensis* (Primulaceae), a new species from Hunan, China. PLoS ONE 10(7): e0132713. 10.1371/journal.pone.0132713 PMC4511667 26201028

